# Research in Medical School: A Survey Evaluating Why Medical Students Take Research Years

**DOI:** 10.7759/cureus.741

**Published:** 2016-08-18

**Authors:** Akhilesh S Pathipati, Noushafarin Taleghani

**Affiliations:** 1 School of Medicine, Stanford University School of Medicine; 2 Department of Emergency Medicine, Stanford University School of Medicine

**Keywords:** medical education, research, medical school

## Abstract

Introduction: In recent years, an increasing number of medical students have taken time off during medical school in order to conduct research. Schools and students have invested millions of dollars and thousands of person-years on research projects, but little is known as to why students choose to take this time off. We aim to characterize why students take research years during medical school.

Methods: The authors distributed an online survey about research in medical school to students at five medical schools that have highly regarded research programs.

Results: 328 students responded to the survey. The most common reasons students take years off for research are: “increase competitiveness for residency application” (32%), “time to pursue other opportunities” (24%), and “academic interest” (23%). Students who would still take a research year even if they were already assured a position in a residency program of their choice were at 65%, while 35% would not take a research year. Responses varied based on whether students intended to go into a competitive specialty.

Discussion: Medical students take research years for multiple reasons, although they frequently are not motivated by an interest in the research itself. Many student projects consume a substantial amount of time and money despite having little educational value. Medical schools, residency programs, and policymakers should rethink incentives to increase value and help students better pursue their academic interests.

## Introduction

Research opportunities are ubiquitous in American medical training. Medical schools offer several reasons for this emphasis. First, schools want to prepare students for careers in academic medicine, and research introduces them to the process of scientific discovery [1]. Another commonly cited reason is that participation in research projects equips students with the critical thinking and analytical skills necessary to stay up-to-date with rapidly evolving literature [2-3].

The evidence is mixed on whether or not medical student research accomplishes these goals [4-5]. Parsonnet et al., note that there are no data to suggest medical student research improves students’ skills as clinicians or academicians [6]. Amgad et al., conducted a systematic review and meta-analysis showing that doing research as a medical student is correlated with pursuing a career as an academic physician; although the authors acknowledge that there is limited evidence, and the lack of prospective studies makes it impossible to establish causality [3]. Bierer and Chen point out that studies on the topic rely heavily on descriptive, self-reported data, preventing any definitive conclusions [7].

Regardless, schools have increasingly invested time and resources in developing research offerings [3-6]. According to a Liasion Committee on Medical Education (LCME) survey in 2012-13, 49 out of 136 medical schools have a research requirement for MD students, some of which consume a full year [8-9]. A sizable majority of schools offer elective research opportunities, and 68% formally support students who want to take a non-degree research year [10]. Medical schools invest an average of $111 million on medical research, in addition to the more than $6 billion distributed to medical schools and teaching hospitals by the National Institutes of Health (NIH) [11].

Students have taken advantage of these opportunities. Data from the American Association of Medical Colleges (AAMC) shows that the percentage of students pursuing non-degree research years has more than doubled in the past 15 years. Just 81% of MD-only students matriculating in 2009 graduated in 4 years, the lowest percentage ever [12].

However, almost no data are available as to why medical students choose to take time off for research. Some students undoubtedly plan to pursue a career as a physician-scientist. For them, a research year is a time to gain experience without having to acquire an MD-PhD.

For others, taking a research year is motivated by different reasons. Research is an important factor in the residency selection process, particularly for competitive specialties [13]. Students may take time off for research in order to increase the competitiveness of their application. Students might also decide to take a research year in order to make time for other pursuits or simply to take a break.

This survey characterizes why medical students take research years. The United States faces a looming physician shortage [14], high rates of physician burnout [15], and the most expensive health care system in the world [16]. Given this context, optimizing physician training and the physician workforce has never been more important. Understanding how students make decisions about their education can help schools to better direct resources to train the next generation of doctors.

## Materials and methods

### Survey design

**Figure 1:**
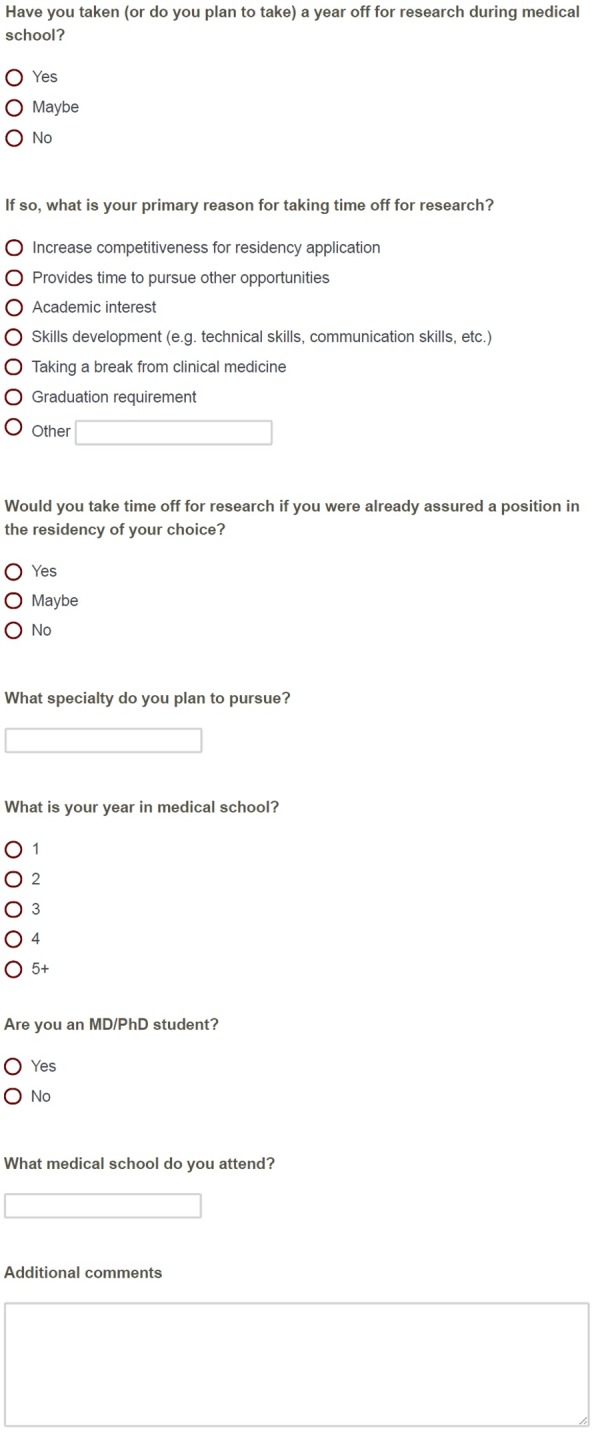
Research Year Survey

Figure 1 depicts the eight-question survey administered to participants. We used the Qualtrics survey tool (Qualtrics; Provo, Utah). The survey was designed to prioritize simplicity. Survey items were constructed based on the author’s experience as a medical student and discussions regarding common reasons students take time off for research.

### Study population

The survey was sent to medical students enrolled at five schools: Stanford University School of Medicine, Harvard Medical School, Johns Hopkins School of Medicine, Perelman School of Medicine at the University of Pennsylvania, and Yale School of Medicine. These five schools were selected because all five are highly regarded research centers that prioritize and financially support student research projects. All of them provide students with resources to take a year off to conduct research. As such, they provide a useful sample of students who are deeply ingrained in the medical school research culture.

### Survey implementation

The survey was distributed to the student body at each of the five selected schools. At Stanford, Johns Hopkins, the University of Pennsylvania, and Yale, students were invited to participate in the survey by an email sent over student listservs. At Harvard, an email listserv was not accessible so the survey was distributed over designated medical student Facebook groups. The survey was open to all enrolled students in the MD program or in a dual-degree program that includes an MD.

### Analysis

Data were compiled and analyzed using Microsoft Excel (Microsoft; Redmond, Washington). We assessed descriptive statistics based on survey responses.

## Results

A total of 328 medical students responded to the survey. Of these, 20 students were MD-PhD candidates. We excluded MD-PhD students from our analysis because these students have a designated emphasis on research for their training and likely have different research priorities than the rest of the medical student population.

This left 308 responses. Table 1 shows the proportion of survey respondents by year in medical school. Third-year students were the most frequent respondents, although there was a spread across all medical school years.

**Table 1:** Number of Respondents by Year in Medical School

Year	%
1	23%
2	14%
3	33%
4	21%
5+	10%

Of the 308 respondents, 186 responded “yes,” 64 responded “maybe,” and 58 responded “no” to the question: “Have you taken (or do you plan to take) a year off for research during medical school?”

We evaluated whether or not there was a difference in students’ plans to take a research year based on the competitiveness of their chosen specialty. Depending on their response to “What specialty do you plan to pursue?”, we sorted students into three categories: undecided, standard competitiveness, and high competitiveness. Students who did not indicate a particular specialty choice were placed in the “undecided” category (n=64). We used USMLE Step 1 scores as a proxy to determine the competitiveness of different specialties. Students who indicated that they planned to pursue a specialty with an average Step 1 score < 240 as per the 2014 National Resident Matching Program data [17] were sorted into “standard competitiveness” (n=103), and students planning to pursue a specialty with an average Step 1 score > 240 were sorted into “high competitiveness” (n=85).

Survey respondents entering “high competitiveness” fields who answered “yes” or “maybe” in regard to taking a year off for research were 92%, compared to 75% of those entering “standard competitiveness” specialties (statistically significant on chi-square testing, p=0.0009), and 81% of those who were undecided (p=0.029).

We then looked further into students’ motivation for taking research years. We limited this analysis to those students who responded “yes” or “maybe.” This left 250 responses.

**Figure 2:**
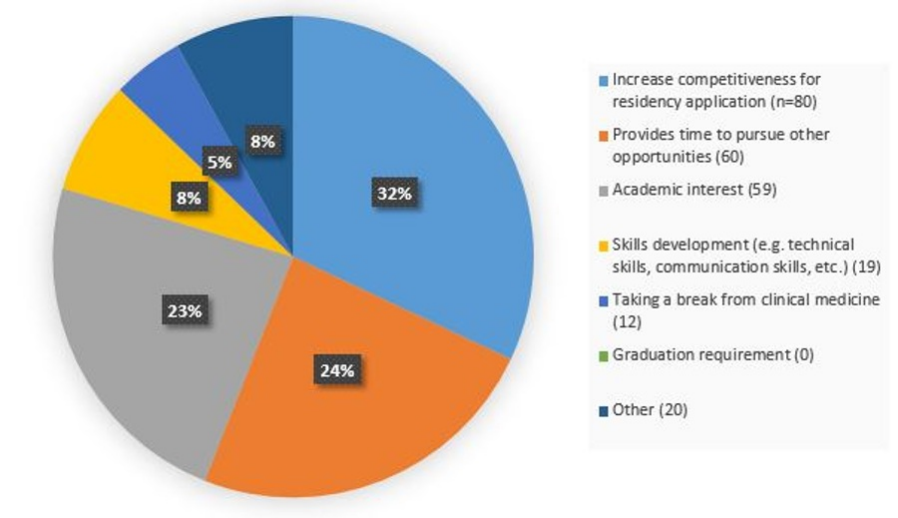
Primary Reason for Taking a Year Off for Research

Figure 2 shows responses to “What is your primary reason for taking time off to do research?” The most frequent response was “increase competitiveness for residency application” (n=80), while “provides time to pursue other opportunities” (n=60) and “academic interest” (n=59) were also common responses. Many respondents who replied with “Other” (n=20) as their reason for taking a research year did so for family reasons. Three respondents stated that they wanted to take time to have a child, while a few mentioned that taking a research year would help align their schedule with that of a significant other. Several respondents took time off for research because they hadn’t yet decided on a specialty and wanted more time to think about it.

**Figure 3:**
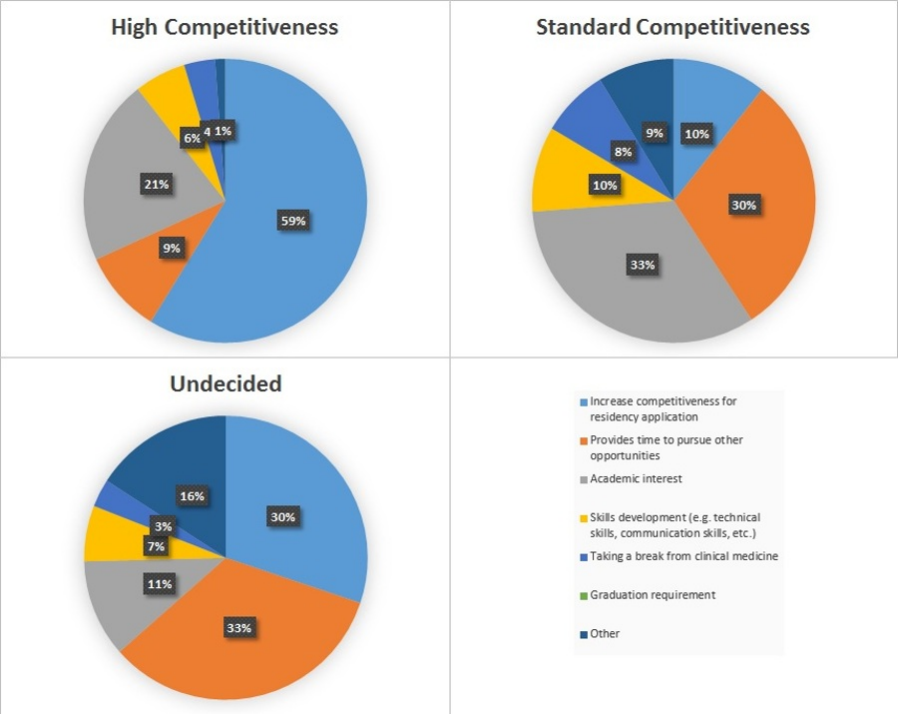
Primary Reason for Taking a Year Off for Research by Specialty Choice

Student responses were considerably different depending on specialty choice. Figure 3 shows the response breakdown by planned specialty. Of note, 59% of respondents going into highly competitive specialties took a year off for research to increase the competitiveness of their application, while just 10% of those going into standard competitiveness specialties did so.

**Figure 4:**
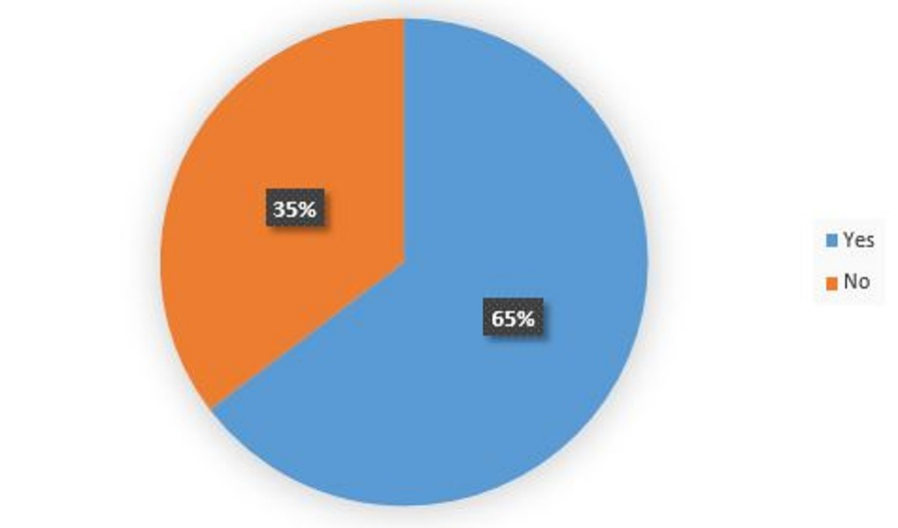
Taking a Research Year if Residency Position Was Already Assured

When asked if they would take a research year if already assured a position in the residency of their choice, 65% of the respondents said "yes" (n=161), while 35% said "no" (n=88) (Figure 4). However, only 41% of those going into competitive fields stated that they would still do a research year in this scenario, whereas 86% of those going into less competitive fields and 59% of undecided students would still do so.

46% of respondents (n=115) indicated that they plan to conduct research as part of their career, 48% responded “maybe” (n=120), and 6% said “no” (n=15). 

## Discussion

This survey represents the first effort to characterize why medical students take years off for research. Medical schools publicize research opportunities and strongly encourage students to take advantage of them, but little is known about why students choose to do so.

We found that although many students (23%) consider “academic interest” to be their primary reason for taking time off for research, these students are a distinct minority. Other factors drive the decision-making of most students. Moreover, students’ motivation varies depending on the competitiveness of their desired specialty.

### The role of residency admissions

“Increasing competitiveness for residency application” was the most commonly cited reason students gave for taking a research year (32%). Research plays a crucial role in the selection process for many residency programs, and it has only become more important over time. Applicants matching in plastic surgery or radiation oncology averaged more than 12 publications in medical school in 2014 (including posters and presentations) [17]. Several fields boast of similarly high publication numbers for matched applicants. Unfortunately, this creates incentives for students to conduct research even if they are not intrinsically interested in doing so. Our survey suggests that this is often the case.

This trend is especially apparent for those students interested in competitive specialties. 59% of students going into “high competitiveness” specialties indicated that they were doing a research year primarily to increase their competitiveness for residency. The majority would not have done so if they did not have to worry about residency admissions. Our results leave little doubt that students consider research to be a key differentiator that residency programs use in selecting candidates.

There are several consequences to this incentive system. One, it pushes students to prioritize quantity over quality when conducting research. Wickramasinghe et al., note an exponential rise in medical student publications between 1980-2010, with the majority coming from medical students in the United States [18]. However, most of these studies are never cited.

In other cases, research incentives lead students to misrepresent their work. Grimm and Maxfield evaluated the publication rate of unpublished manuscripts submitted to a radiology residency program via the Electronic Residency Application Service [19]. They found that approximately one-third of manuscripts listed as “accepted,” “in press,” or “provisional accepted” remained unpublished two years later.

### Research as time to pursue other opportunities

A large proportion of survey respondents indicated that their research time was primarily intended as “time to pursue other opportunities” (24%). Medical students are bright and capable individuals with diverse interests. These include policy projects, technology, community health initiatives, and a host of personal hobbies. For many of them, taking a research year is a way to obtain funding while pursuing their own interests. For example, one student stated that “My ‘research year’ was for writing.”

While these students are on a “research year,” their primary interest lies elsewhere. However, medical schools have few formal opportunities built to provide students with exposure to other aspects of medicine and health care. Students often seek those out on their own, but are required to go through the motions of conducting research, which requires a substantial amount of time and effort and detracts from their actual goals.

### Other responses

A smaller proportion of respondents indicated that they took their research year for “skills development,” “taking a break from clinical medicine,” and “other” reasons. A unifying theme across these responses was the notion of taking a research year for personal reasons rather than academic ones. In some cases, that meant personal development through skills acquisition. For others, it was for wellness and a break from the grind of medical school. As one student put it, “I was abused and bullied during MS3 and needed time for psychological recovery.”

### Rethinking medical student research

It is a central tenet of American medical education that students should conduct research It is time to start questioning that dogma. There is little reason to believe medical student research improves students’ abilities to take care of patients [4, 20], and evidence is equivocal at best on whether early exposure to research leads students to become physician-scientists [3, 7].

This study indicates that students who pursue research years often do so despite a lack of interest. Over one-third of survey respondents who have taken or may take a research year indicated that they would not do so if residency were not a consideration. For those who would still take a year off for research, it is often so that they can pursue non-research activities. Perhaps this is why the research produced by students is often low quality and contributes little original knowledge [18].

In spite of these findings, the number of medical students taking time off for research continues to rise. Every year, millions of dollars and hundreds of person-years are spent on activities that lack educational value, do not contribute to society, and that students do not want to do. Some of the most talented young people in the country spend their time generating papers that no one will read. The medical system cannot afford this waste.

This brings up the following two questions. First, why does the problem exist? And second, what can be done about it? These questions lie outside the scope of this paper, but medical schools, residencies, and the academic medical enterprise should invest in answering them.

It is worth noting that the vast majority of survey respondents expressed an interest in pursuing research as part of their careers. What is more, many students have interesting ideas for projects. They are interested in scholarly work, but are too often doing it for the wrong reasons. Students have the potential to bring innovative thinking to some of the most significant challenges in healthcare and medicine, but instead prioritize small projects that are more likely to result in a publication. There is a profound need to realign incentives in medical education.

### Limitations of the study

This study had several limitations. First, as with any survey, we are limited by our response rate. A sizable number of students responded to the survey, but these represent a fraction of those to whom it was sent. Second, the survey was only distributed at five schools; all of which are research intensive academic centers. The nature of the responses would likely vary at other institutions. Third, the survey had a relatively narrow scope. We only sought to define why medical students take years off for research. We did not specifically determine if students would prefer alternatives to the status quo or identify what they would like those alternatives to be.

### Future directions

More investigation is needed on several aspects of medical student research. This includes characterizing medical student research years on a national basis and delving deeper into what alternatives medical students would consider. We also believe more information is needed on the nature and quality of medical student research. Finally, as alluded to previously, further consideration should be given as to why research incentives exist in their current form and how they can be modified. The education system must evolve to better harness the potential of medical students.

## Conclusions

Medical students take research years for multiple reasons, although they frequently are not motivated by an interest in the research itself. Many student projects consume a substantial amount of time and money despite having little educational value. Medical schools, residency programs, and policymakers should rethink incentives to increase value and help students better pursue their academic interests.

## References

[1] Small AC, Levy LL (2013). In support of medical student research. Acad Med.

[2] Solomon SS, Tom SC, Pichert J, Wasserman D, Powers AC (2016). Impact of medical student research in the development of physician-scientists. J Investig Med.

[3] Amgad M, Man Kin Tsui M, Liptrott SJ, Shash E (2015). Medical student research: an integrated mixed-methods systematic review and meta-analysis. PLoS One.

[4] Chang Y, Ramnanan CJ (2015). A review of literature on medical students and scholarly research: experiences, attitudes, and outcomes. Acad Med.

[5] Rosenkranz SK, Hu W (2016). More on promoting medical student scholarly research. Acad Med.

[6] Parsonnet J, Gruppuso PA, Kanter SL, Boninger M (2010). Required vs. elective research and in-depth scholarship programs in the medical student curriculum. Acad Med.

[7] Bierer SB, Chen HC (2010). How to measure success: the impact of scholarly concentrations on students—a literature review. Acad Med.

[8] Liasion Liasion (2016). Medical student research requirement. Medical.

[9] Laskowitz DT, Drucker RP, Parsonnet J, Cross PC, Gesundheit N (2010). Engaging students in dedicated research and scholarship during medical school: the long-term experiences at Duke and Stanford. Acad Med.

[10] (2016). Student research year questionnaire. Student.

[11] Association Association (2016). Academic medicine investment in medical research. Academic Medicine Investment.

[12] Association Association (2016). Graduation rates and attrition factors for U.S. medical school students. Graduation.

[13] (2016). Results of the 2014 NRMP program director survey. the.

[14] Association Association (2016). Physician supply and demand through 2025: key findings. Physician.

[15] Shanafelt TD, Boone S, Tan L (2012). Burnout and satisfaction with work-life balance among US physicians relative to the general US population. Arch Intern Med.

[16] Davis K, Stremikis K, Squires D, Schoen C (2016). Mirror, Mirror on the Wall: How the Performance of the U.S. Health Care System Compares Internationally. Accessed.

[17] (2016). Charting outcomes in the match. Accessed.

[18] Wickramasinghe DP, Perera CS, Senarathna S, Samarasekera DN (2013). Patterns and trends of medical student research. BMC Med Educ.

[19] Grimm LJ, Maxfield CM (2013). Ultimate publication rate of unpublished manuscripts listed on radiology residency applications at one institution. Acad Med.

[20] Emanuel EJ, Fuchs VR (2012). Shortening medical training by 30%. JAMA.

